# COVID-19-Induced Takotsubo Cardiomyopathy With Concomitant Pulmonary Embolism

**DOI:** 10.7759/cureus.18693

**Published:** 2021-10-11

**Authors:** Lalith Namburu, Sukhdeep s Bhogal, Vijay K Ramu

**Affiliations:** 1 Internal Medicine, East Tennessee State University Quillen College of Medicine, Johnson City, USA; 2 Cardiology, East Tennessee State University Quillen College of Medicine, Johnson City, USA

**Keywords:** takotsubo cardiomyopathy, covid-19, saddle pulmonary embolism, acute hypoxic respiratory failure, sars-cov-2

## Abstract

Coronavirus disease 2019 (COVID-19), which is caused by severe acute respiratory syndrome coronavirus 2 (SARS-CoV-2), has emerged as a global pandemic with an unprecedented death toll worldwide. Although it primarily affects the respiratory tract presenting as pneumonia or acute respiratory failure, it is also known to cause significant cardiovascular complications, including acute coronary syndrome (ACS), arrhythmia, myopericarditis, cardiomyopathy, venous thromboembolism, heart failure, and cardiogenic shock. Morbidity and mortality secondary to cardiovascular complications are higher in patients with preexisting cardiovascular risk factors. Here, we present a case report of a 69-year-old male who was recently diagnosed with COVID-19 illness presenting with ST-elevation myocardial infarction (STEMI) and eventually with Takotsubo cardiomyopathy (TTC), and the course was complicated by right atrial thrombus and a pulmonary embolism (PE).

## Introduction

Takotsubo cardiomyopathy (TTC), also known as apical ballooning syndrome, broken heart syndrome, or stress-induced cardiomyopathy, is caused by intense emotional or physical, or metabolic stressors and is characterized by transient, often with reversible, left ventricular systolic dysfunction usually in the absence of obstructive coronary disease on coronary angiography. TTC has been identified as one of the cardiovascular complications of coronavirus disease 2019 (COVID-19) [1]. Also, COVID-19 is known to cause a hypercoagulable state and can present with pulmonary embolism (PE). However, simultaneous presentation of TTC and PE has been a rare entity, and no case reports are published to date.

## Case presentation

A 69-year-old Caucasian male with a history of hypertension presented to the hospital with chest pain and dyspnea for the last week. He described the chest pain as substernal, pressure-like, and unrelated to activity with worsening over a couple of days. The patient was recently diagnosed with COVID-19 illness 10 days prior to the presentation. The vitals in the emergency room (ER) were as follows: blood pressure of 132/88 mmHg, heart rate of 124 beats/minute, respiratory rate of 28 breaths/minute, oxygen saturation of 83% on four-liter oxygen, and temperature of 98.8°F. Physical examination was significant for moderate to severe respiratory distress with bilateral basilar crackles and sinus tachycardia. He was placed on a high-flow nasal cannula with six-liter oxygen, and his oxygen saturation improved to 95%.

**Figure 1:**
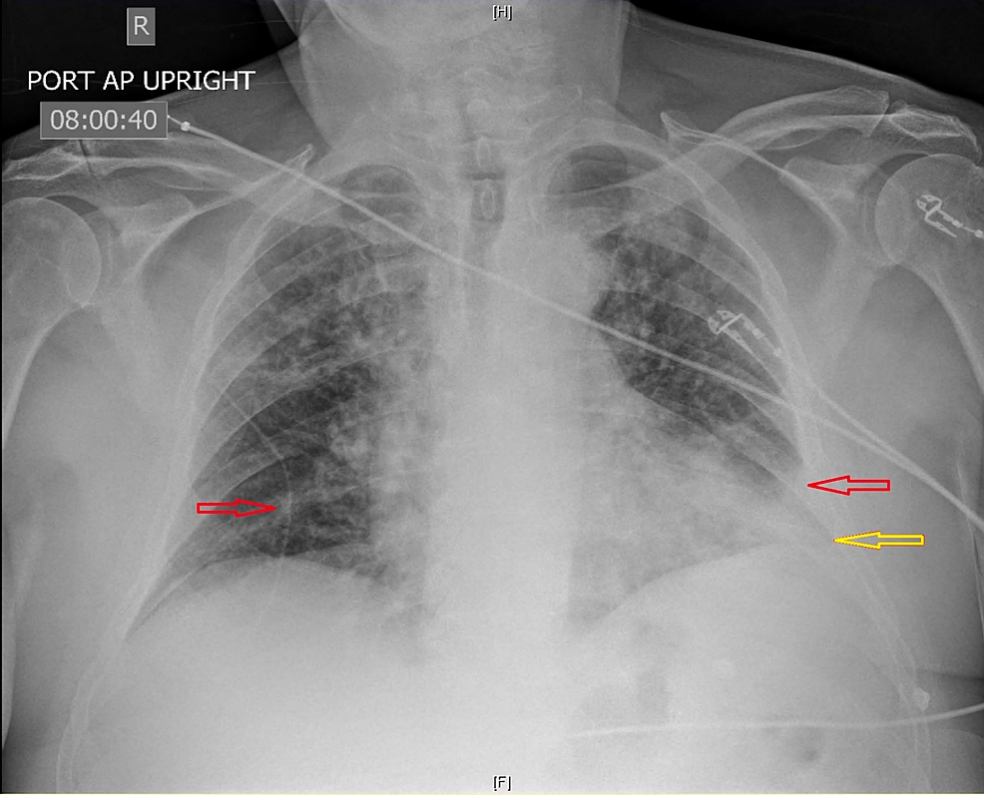
Chest X-ray showing bilateral basal patchy opacities (red arrows) and left-sided pleural effusion (yellow arrow).

**Figure 2:**
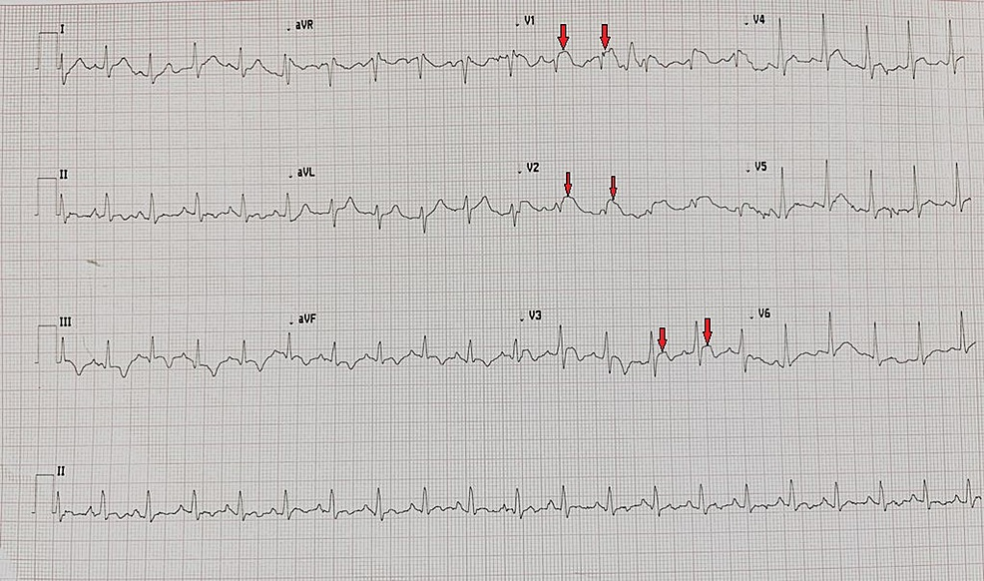
ECG showing ST elevations in V1–V3 leads (red arrows).

Chest X-ray demonstrated bilateral patchy opacities most prominent at left lung base with minimal left pleural effusion (Figure 1). His electrocardiogram (ECG) in the ER showed ST elevations in V1-V3 leads consistent with the diagnosis of ST-elevation myocardial infarction (STEMI) (Figure 2). The laboratory test results were significant for leukocytosis (white blood cell count: 22.4 K/µL) with neutrophilia (absolute neutrophil count: 20.1 K/µL), high troponin I (4.99 ng/mL), and high inflammatory markers (C-reactive protein [CRP]: 170.4 mg/L; ferritin: 890 ng/mL). The patient was retested for severe acute respiratory syndrome coronavirus 2 (SARS-CoV-2), which was positive.

**Figure 3:**
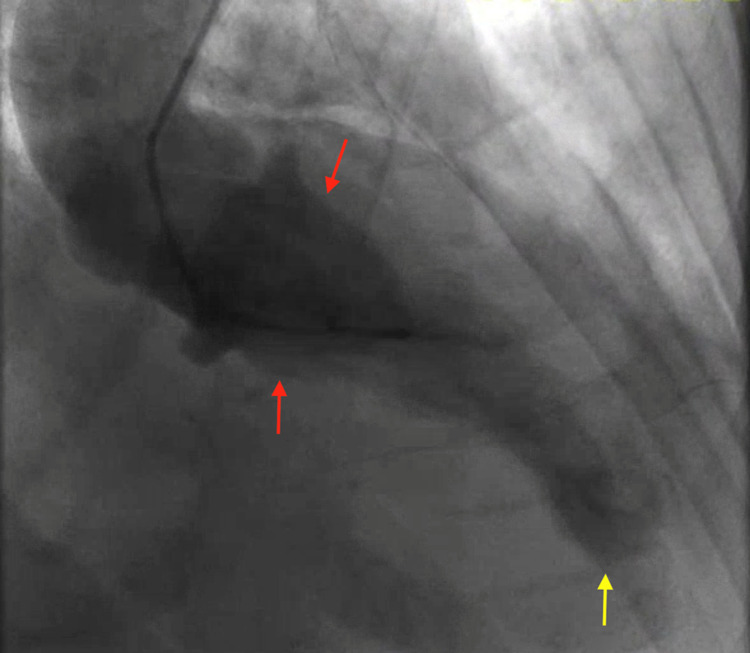
Left ventriculogram demonstrating akinesis of the apical left ventricle wall (yellow arrow) and basal hyperkinesis (red arrows) consistent with Takotsubo cardiomyopathy.

**Figure 4:**
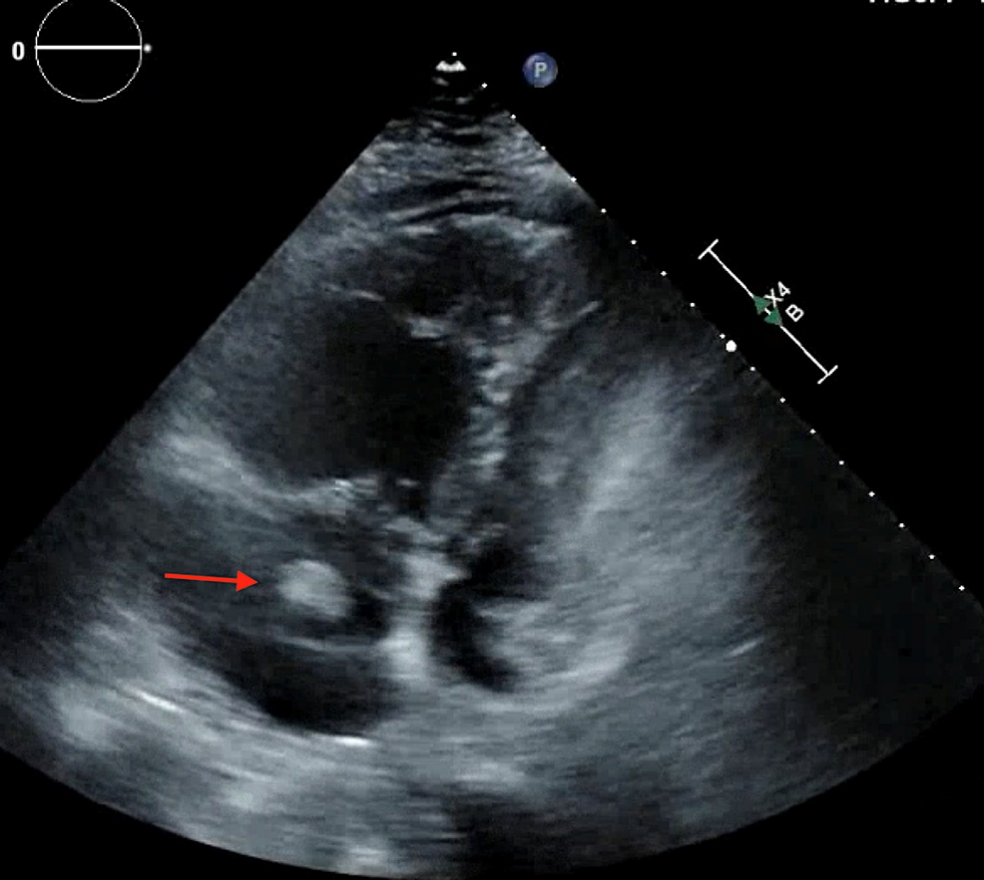
Transthoracic echocardiogram showing a thrombus in the right atrium (red arrow) and dilated right ventricle (apical four-chamber view).

**Figure 5:**
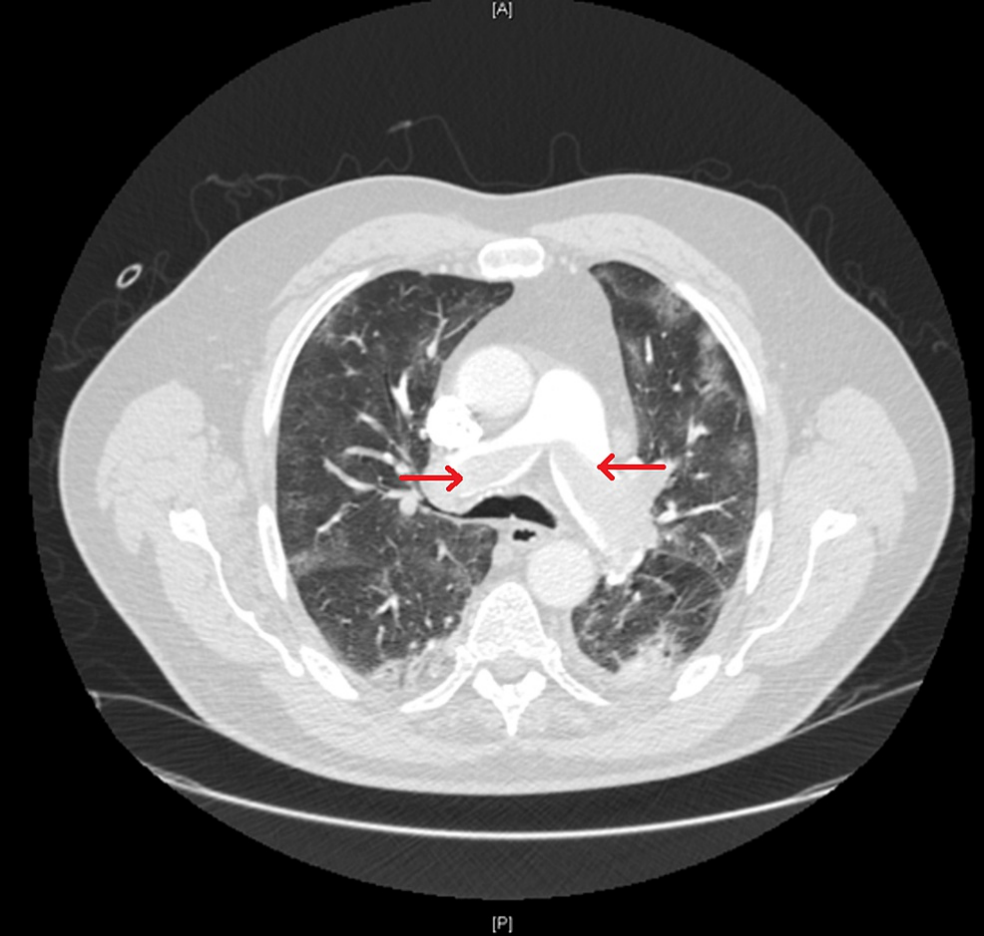
Computed tomography demonstrating saddle pulmonary embolism at the bifurcation of the pulmonary trunk (red arrows).

**Figure 6:**
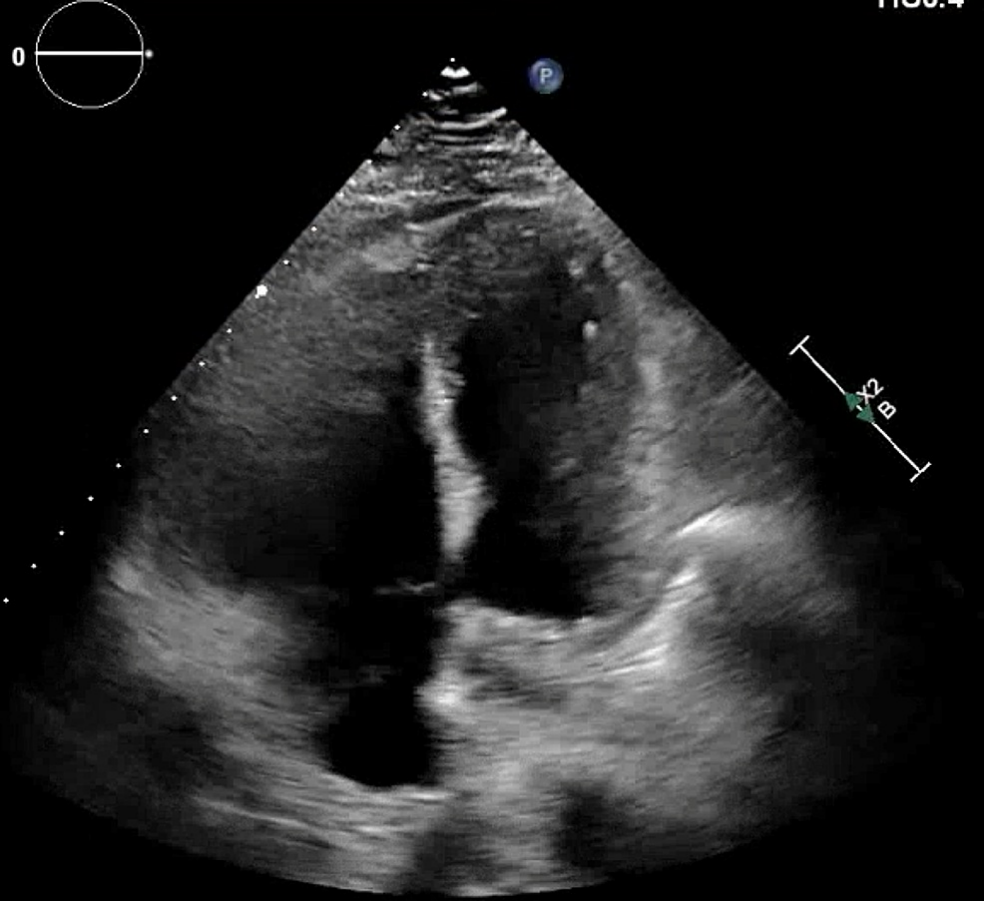
Follow-up transthoracic echocardiogram showing thrombus resolution in the right atrium (apical four-chamber view).

He was emergently taken to the cardiac catheterization laboratory and underwent left heart catheterization (LHC) showing nonobstructive coronary artery disease with apical ballooning of the left ventricle, a ventriculogram characteristic of TTC (Figure 3). Transthoracic echocardiogram (TTE) showed a left ventricular ejection fraction (LVEF) of 45%, enlarged right ventricle (RV), and a right atrial thrombus (Figure 4). Given a high index of suspicion for a pulmonary embolism, a computed tomography angiogram (CTA) with contrast was obtained and showed a large burden of bilateral PE with right heart strain (Figure 5). The patient underwent thrombolysis and was monitored closely in the intensive care unit. The patient was started on supportive care with aspirin, lisinopril, metoprolol succinate, and Lasix for TTC along with anticoagulation for PE. TTE obtained a week later showed improvement in LVEF (60%) with resolution of right atrial thrombus (Figure 6). The patient’s acute hypoxic respiratory failure improved eventually, the rest of the hospital course was uneventful, and the patient was subsequently discharged.

This case report highlights cardiovascular insults secondary to COVID-19 that can complicate the disease course. It is important to have cardiovascular complications of COVID-19 as a differential diagnosis at the time of presentation for prompt diagnosis and treatment.

## Discussion

COVID-19 illness is known to primarily affect the respiratory system with patient presentation varying from asymptomatic infection to severe pneumonia causing acute respiratory failure and acute respiratory distress syndrome [2]. It is increasingly evident that it can affect multiple organ systems and can present with a plethora of symptoms and that physicians must have a high suspicion for COVID-19 to diagnose and prevent mortality and morbidity with the disease. A high incidence of cardiac injury (up to 20%) has been reported in these patients, and multiple mechanisms are being suggested for cardiovascular insult [3]. These mechanisms include direct cardiotoxicity, hypoxemia-mediated injury, supply-demand mismatch, cytokine storm, and vascular endothelial dysfunction. The above mechanisms can cause ischemic myocardial injury, nonischemic myocardial injury (myocarditis, TTC), arrhythmia, and venous thromboembolism [2].

TTC, also known as apical ballooning syndrome, broken heart syndrome, or stress-induced cardiomyopathy, is caused by intense emotional or physical, or metabolic stressors and is characterized by transient, often with reversible, left ventricular systolic dysfunction usually in the absence of obstructive coronary disease on coronary angiography [4]. TTC has been identified as one of the cardiovascular complications of COVID-19 [5]. Also, COVID-19 is known to cause a hypercoagulable state and can present with pulmonary embolism [6], as in our case. However, the simultaneous presentation of TTC and PE seems to be a rare entity, and no case has been reported so far in the literature. 

Although the exact underlying pathophysiology of TTC is unknown, it is widely accepted to be catecholamine-induced myocardial stunning [7]. The COVID-19 disease causes cytokine release syndrome characterized by high levels of circulating catecholamines eventually leading to TTC [8]. Our patient also had intracardiac thrombus on TTE, and subsequent CTA showed bilateral PE with right heart strain. Considering that the acute hypoxic respiratory failure was likely from PE, the patient underwent thrombolysis and anticoagulation, leading to improvement in respiratory symptoms and resolution of right atrial thrombus. Interestingly, a recent review has shown that COVID-19-related TTC has a higher incidence in men compared with women [5]. To the best of our knowledge, our case is the first in the literature reporting COVID-19-induced TTC with concomitant PE.

## Conclusions

COVID-19-induced TTC and concomitant PE is a rare entity. It is important to keep cardiovascular manifestations of COVID-19 in the differentials at the time of patient presentation. TTC has been well documented to be associated with COVID-19, and we believe that the incidence could be increasing with COVID-19 given both physical and emotional stress with this illness.
